# Antioxidants as an Epidermal Stem Cell Activator

**DOI:** 10.3390/antiox9100958

**Published:** 2020-10-07

**Authors:** Soon-Hyo Kwon, Kyoung-Chan Park

**Affiliations:** 1 Department of Dermatology, Kyung Hee University Hospital at Gang-dong, Kyung Hee University School of Medicine, Seoul 05278, Korea; soonhyo17@hanmail.net; 2 Department of Dermatology, Seoul National University College of Medicine, Seoul National University Bundang Hospital, Gyeonggi 13620, Korea

**Keywords:** antioxidants, epidermal stem cell, extracellular matrix, niche, skin equivalent

## Abstract

Antioxidants may modulate the microenvironment of epidermal stem cells by reducing the production of reactive oxygen species or by regulating the expression of extracellular matrix protein. The extracellular membrane is an important component of the stem cell niche, and microRNAs regulate extracellular membrane-mediated basal keratinocyte proliferation. In this narrative review, we will discuss several antioxidants such as ascorbic acid, plant extracts, peptides and hyaluronic acid, and their effect on the epidermal stem cell niche and the proliferative potential of interfollicular epidermal stem cells in 3D skin equivalent models.

## 1. Introduction

Epidermis, a multilayered tissue, is mainly composed of renewable keratinocytes. Basal keratinocytes proliferate and migrate into the upper layer of the epidermis. They undergo the process of differentiation thereafter in which their cuboidal shape becomes flatten. At the end of their fate, keratinocytes finally lose their nuclei and are eliminated from the skin. Epidermal stem cells or progenitor cells with high proliferative ability contribute to the maintenance of skin through their self-renewal, proliferation, and differentiation capacities [1]. The proliferation and survival of epidermal stem cells is influenced by numerous intrinsic and extrinsic factors [2]. A reduction in the function or number of stem cells may cause impaired self-renewal of the skin, thereby resulting in skin aging [3]. Senescence of keratinocytes by chronological aging or repetitive subculture causes a reduction in stem cell population in vitro [4]. The epidermal stem cells are located in a ‘niche’—specific sites that determine stem cell activity and homeostasis [5]. Stem cell niche provides a stem cell-specific microenvironment. As a result, it regulates the number and function of stem cells, and thus, it is important for the fate of stem cells [6].

Our team has studied the effect of antioxidants on the interfollicular epidermal stem cells [7,8,9,10,11,12]. Well-known antioxidants such as ascorbic acid, resveratrol and copper-GHK, as well as plant extracts that have been used for anti-fatigue or anti-stress treatment in traditional medicines have been evaluated for their role to activate the proliferative potential of epidermal stem cells. In this narrative review, we will discuss the importance of redox balance in the regulation of epidermal stem cells. In vitro and in vivo study results that demonstrate the antioxidant activities of the molecules such as ascorbic acid, plant extracts (*Ganoderma lucidum*, *Rhodiola*, resveratrol, *Eleutherococcus senticosus*), tripeptides, and hyaluronic acid will be overviewed. Experimental data supporting the role of the antioxidants on modulating stem cell microenvironment and stimulating the proliferative potential of epidermal stem cells will be reviewed. Finally, we will discuss the applicability of the antioxidants for anti-aging treatment of skin.

## 2. Epidermal Stem Cells and Extracellular Matrix

### 2.1. Interfollicular Epidermal Stem Cells

Three distinct epidermal stem cell niches have been demonstrated—bulge of hair follicle, base of sebaceous gland, and basal layer of interfollicular epidermis [13]. Contrary to hair follicular stem cells, little is known about markers for the interfollicular epidermal stem cells. p63 is a putative marker for the interfollicular epidermal stem cells that is an essential transcription factor for epidermal development and homeostasis [14,15,16]. It determines fate of the keratinocytes by regulating the balance between stemness, differentiation, and senescence [15]. However, it is not a specific marker, as differentiated keratinocytes in the upper epidermis also expresses p63. Thus, other markers, such as proliferating cell nuclear antigen (PCNA) that is involved in the cell cycle in proliferating cells, have been used to identify interfollicular epidermal stem cells [17]. Recently, we reported a new method to identify interfollicular epidermal cells by combining the expressions of p63 and histone deacetylase (HDAC) 1 [13].

### 2.2. Extracellular Matrix and Stem Cell Niche

Extracellular matrix (ECM) is an important component of the stem cell niche [18]. High levels of integrin have been proposed to be a marker for epidermal stem cells [19]. By expressing high levels of integrins, epidermal stem cells can interact with the ECM. Integrin α6 is a marker of extracellular adhesion receptors that exist along the dermo-epidermal junction [20]. Integrin β1 is expressed on the membrane of basal keratinocytes and mediates the cell-to-matrix and cell-to-cell interactions [21]. Both of these integrins are basement membrane proteins related to the stemness of the epidermis [22].

We previously reported that insulin-like growth factor-binding protein (IGFBP)-2 from hair follicle dermal sheath cells plays a critical role in regulating regenerative capacity of human epidermal keratinocytes [23]. Skin equivalents treated with IGFBP-2 showed thicker and more matured epidermis than control. The expressions of integrin α6 and β1 were upregulated when compared with control. IGFBPs form biologically active complexes with insulin growth factor (IGF)-1 and vitronectin [24,25]. Through the activation of both the IGF receptor and vitronectin-binding integrins, IGF-1 could protect keratinocytes against UVB damage [24]. Furthermore, activation of integrin β1 is involved in the inhibition of differentiation signals via extracellular signal-regulated kinases (ERK) signaling pathway [26]. These findings suggest integrins are important in regulating stem cell physiology and determining the fate of epidermal stem cells in skin [11]. 

Recently, we reported the relationship between ECM production and epidermal stem cell function via an epigenetic mechanism [27]. MicroRNA (miR135b), which regulates the synthesis of type IV collagen, the major component of cutaneous basement membrane, is involved in the maintenance of epidermal stem cell niche and proliferative potential of epidermal basal cells. Inhibition of miR135b increased the number of p63- and PCNA-positive cells in the epidermis in vitro via enhanced expression of type IV collagen. 

### 2.3. Reactive Oxygen Species Regulates Stem Cell Niche

Since skin constitutes the outer surface of human body that is constantly exposed to environmental stimuli, the epidermal stem cells are regulated by not only the interaction with intrinsic transcriptional regulations but also extrinsic environmental signals [11]. These extrinsic factors could affect stem cell microenvironment, altering the fate of epidermal stem cells [28]. For example, hypoxia is required for the maintenance of undifferentiated stem cell and for controlling the proliferation and survival of stem cells [29]. Reactive oxygen species (ROS) are important in the stem cell development, function, and survival [30]. ROS enhance differentiation of human embryonic stem cells into mesendodermal lineage via ROS-involved signaling pathways [31]. Coenzyme Q10, a well-known antioxidant, has been shown to have protective effect against hypoxia-reperfusion induced damage in neural stem cells [32]. These findings indicate that redox status is critical in regulating stem cells. 

ROS modulate the microenvironment surrounding epidermal stem cells, affecting their proliferative potency [7]. It has been shown that epidermal keratinocyte subpopulations with stem-cell like characteristics have lower levels of antioxidant proteins (superoxide dismutase (SOD), catalase, and glutathione peroxidase-1), and decreased activity of SOD and catalase compared with more differentiated keratinocytes [33]. They also exhibit less mitochondrial area, fewer peroxisomes, and lower levels of ROS production than differentiated keratinocytes. Furthermore, aging causes a reduction in the antioxidant capacity of epidermal keratinocytes as shown by decreased concentrations of α-tocopherol, ascorbic acid, and glutathione [34]. 

### 2.4. UV Radiation Affects Epidermal Stem Cells Via ROS Production

Chronic ultraviolet (UV) radiation is the main cause of extrinsic skin aging or photoaging. UVB (280–320 nm) and UVA (320–400 nm) radiation causes not only direct injury in molecules such as nucleic acids via cyclobutane pyrimidine dimers and pyrimidine (6–4) pyrimidine photoproducts formation, but also oxidative stress and the production of intracellular ROS [35,36,37,38]. ROS are primarily generated within the inner membrane of mitochondria [38]. Electrons leaked from the mitochondrial electron transport process react with oxygen to form free radicals [38]. UV-induced ROS react with lipids, proteins, and DNA, which leads to cellular apoptosis and gene mutations [39]. 

To protect against cellular damage from the UV-induced ROS, epidermal keratinocytes express several antioxidant enzymes, such as SOD, catalase, and glutathione peroxidase-1, that regulate the generation of intracellular ROS [33]. The endogenous antioxidant machinery in the epidermis has higher activity or concentration percentages compared with that in dermis: SOD (126%), glutathione peroxidase (61%), glutathione reductase (215%), glucose-6-phosphate dehydrogenase (111%), isocitrate dehydrogenase (313%), α-tocopherol (90%), ubiquinol 10 (900%), ascorbic acid (425%), uric acid (488%), reduced glutathione (513%), and total glutathione (471%) [40]. UVB, which is the most important environmental factor causing skin photoaging, down-regulates the antioxidant enzyme activity, induces the production intracellular ROS in the epidermis, and thus disrupts the cellular defense system [41,42]. As a result, elevated levels of intracellular oxidized proteins are observed in aged skin [43]. 

## 3. Antioxidants and Interfollicular Epidermal Stem Cells

### 3.1. Skin Equivalents

The effect of the antioxidants on the production of ECM and epidermal stem cells could be evaluated using a 3D skin equivalent model rather than two-dimensional keratinocyte cultures to investigative the interaction between interfollicular epidermal stem cell and ECM. The process of constructing skin equivalents is described in our previous study [44]. Dermal substitutes were produced by mixing type I collagen from the tendons of rattails, 10× concentrated DMEM, and neutralization buffer (0.05 N NaOH, 0.26 mM NaHCO_3_, and 200 mM HEPES), and adding 5 × 10^5^ human fibroblasts. After gelling in a 30 mm polycarbonate filter chamber, 1 × 10^6^ human keratinocytes were seeded onto the dermal substitute. After 24h of submerged culture, they were cultured at the air–liquid interface for twelve days. The growth medium consisted of DMEM and Ham’s nutrient mixture F12 at a ratio of 3:1, 5% FBS, 0.4 μg/mL hydrocortisone, 1 μM isoproterenol, 25 μg/mL ascorbic acid, and 5 μg/mL insulin. EGF with a concentration of 1 ng/mL was added during the submerged culture, and EGF with 10 ng/mL was added during the air–liquid interface culture. 

### 3.2. Ascorbic Acid

#### 3.2.1. Antioxidant Activity

Ascorbic acid is an essential human dietary requirement for the prevention of scurvy. Scurvy is characterized by hemorrhages, wound dehiscence, poor wound healing, and loosening of teeth [45]. Symptoms of scurvy indicate that ascorbic acid is involved in the synthesis of connective tissue. In fact, ascorbic acid catalyzes posttranslational modification of procollagen to produce mature collagen [46]. Hydrolases that convert prolyl and lysyl residues of procollagen to produce stable triple helical structure of collagen require ascorbic acid as a cofactor [46]. 

Ascorbic acid is a well-known antioxidant that reduces intracellular ROS production. It acts as an electron donor to reduce the production of ROS that is generated during metabolic respiration or mitochondrial oxidative phosphorylation [45]. As a result, ascorbic acid protects cells from oxidative DNA damage, lipid peroxidation, and the oxidation of amino acid residues [47]. Several studies have reported that ascorbic acid decreases UVB-induced ROS production, and prevents ROS-medicated DNA damage and apoptosis in epidermal keratinocytes [48,49]. Pre- and post-treatment of reconstituted human epidermis with ascorbic acid reduced UVB-induced cell death, apoptosis, DNA damage, ROS production, and the inflammatory response by suppressing tumor necrosis factor-α (TNF-α) production [50]. 

#### 3.2.2. Epigenetic Regulation

In addition to its role as an antioxidant, ascorbic acid also serves as an epigenetic regulator of histone and DNA methylation. Ascorbic acid acts as a cofactor for Fe^2+^ and α-ketoglutarate-dependent dioxygenases (Fe^2+^/ α-KGDDs) by transferring electron to reduce ferric iron (Fe^3+^) to ferrous iron (Fe^2+^) [51]. Diverse families of Fe^2+^/ α-KGDDs include collagen prolyl hydroxylases, and epigenetic regulators such as the Jumonji C domain-containing histone demethylases (JHDMs), DNA and RNA demethylases of the AlkB homolog family, and the ten-eleven translocation (TET) family of DNA hydroxylases [52,53,54,55,56,57]. By upregulating the activity of JHDMs or TET DNA hydroxylases, ascorbic acid regulates the embryonic stem cell function and promotes reprogramming of fibroblasts to induced pluripotent stem cells (iPSC) [51]. Ascorbic acid was shown to maintain the proliferation of embryonic stem cell and human mesenchymal stem cells in vitro [58,59]. It enhances the activity of JHDM1a/1b to promote demethylation of H3K36me2/3 in mouse embryonic fibroblasts during reprogramming [53]. Ascorbic acid can also induce a specific loss of H3K9me2, which is a barrier during somatic cell reprogramming into iPSCs, in the embryonic stem cells, and drive the transition of pre-iPSC to iPSC by activating JHDMs [58]. Ascorbic acid enhances iPSC reprogramming and DNA demethylation in mouse and human fibroblasts via TET-dependent production of 5-hydroxymethylcystosine [60,61]. 

#### 3.2.3. Epidermal Stem Cell Activation

**Figure 1:**
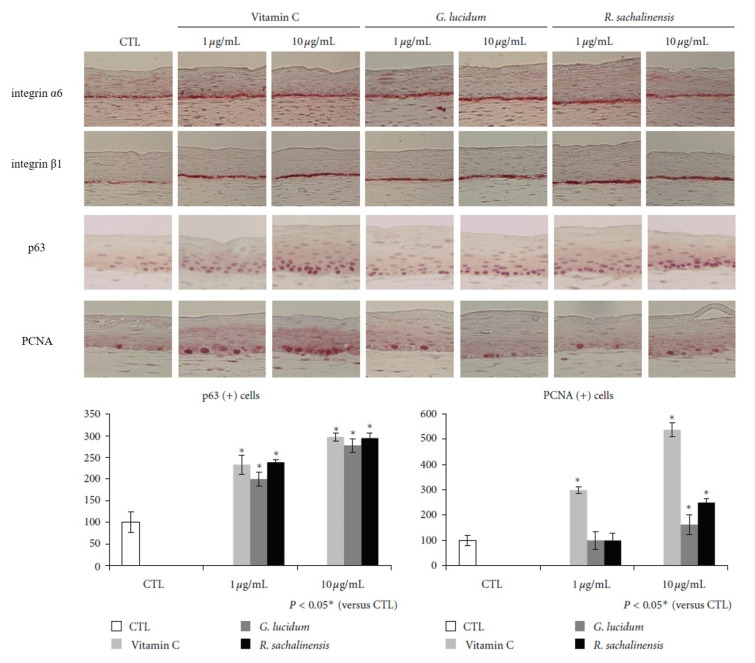
Immunihistochemical staining for integrins, p63, and PCNA. The skin equivalents were constructed and incubated in the presence of vitamin C, *G. lucidum*, or *R. sachalinensis* at 1 or 10 μg/mL. Sections of skin equivalents were stained for integrin α6, integrin β1, p63, and PCNA. Staining intensities of both integrins, p63, and PCNA were significantly increased by addition of vitamin C or plant extracts (magnification x200). (* *p* < 0.05) (modified from the work by Choi et al. [7]).

Ascorbic acid enhances the stemness of corneal epithelial stem cells of mouse by promoting ECM production, and thus accelerates epithelial wound healing in the cornea [62]. An in vitro study revealed that ascorbic acid promotes the proliferation of keratinocytes and fibroblasts from skin [63]. Due to its antioxidant activity and role as a stem cell regulator, we investigated the effect of ascorbic acid on epidermal stem cells [7]. Ascorbic acid showed good antioxidant activity at both low (10 μg/mL) and high concentrations (100 μg/mL) in vitro. More cuboid-shaped basal cells that have high proliferative potential were observed in the skin equivalents treated with ascorbic acid compared with control. The expression of integrins α6 and β1 along the basement membrane was upregulated in the ascorbic acid-treated skin equivalents. (Figure 1) The number of p63- and PCNA-positive cells was increased in skin equivalents treated with ascorbic acid. Thus, by altering the integrin expression, ascorbic acid provides favorable stem cell microenvironment in the skin, increasing the stemness and proliferative potential of epidermal basal cells.

### 3.3. Ganodermal Lucidum

#### 3.3.1. Antioxidant Activity

*Ganoderma lucidum* is a highly nutritional mushroom that has been used in traditional medicines. It is also known as Lingzhi mushroom in China and Reish in Japan. *G. lucidum* has drawn attention as a natural compound with diverse bioactivities, including antioxidant, immunomodulatory, anti-allergic, anti-inflammatory, anti-bacterial, anti-fungal, anti-viral, anti-tumor, and anti-melanogenic activities [64]. Effective components of *G. lucidum* are polyscaccharides glycoproteins, phenols, and protein substances, which provide its antioxidant activity [65]. 

*G. lucidum* polysaccharides, a major component of *G. lucidum*, has a wide spectrum of pharmacologic properties, including antioxidant, anti-aging, anti-tumor, anti-inflammatory, anti-depressant, and immunomodulatory activities [66,67]. *G. lucidum* polysaccharides exhibits antioxidant activity by scavenging ROS and enhancing enzymatic activity of glutathione peroxidase [68,69]. Injection of *G. lucidum* polysaccharides into mice resulted in the decreased low-density lipoprotein (LDL) oxidation products, reduced level of malondialdehyde, and increased glutathione peroxidase activity in serum and heart [68]. *G. lucidum* polysaccharides also scavenged tert-butyl hydroperoxide-induced free radicals in macrophages in vitro and in vivo [69]. By increasing antioxidant enzyme activities and lowering malondialdehyde levels, *G. lucidum* polysaccharides protects murine skeletal muscles from oxidative stress induced by exhaustive exercise [70]. In addition, *G. lucidum* polysaccharides protects brain and renal tissue against ischemia-reperfusion by suppressing oxidative stress and cell apoptosis [71,72,73]. Another *Ganoderma* protein, *Ganoderma* glycopeptides, also exhibits antioxidant property. *Ganoderma* glycoprotein increased the activity of SOD and glutathione peroxidase in animal serum, hippocampus, and myocardium, resulting in the reduced level of malondialdehyde [65]. 

*G. lucidum* polysaccharides have been widely used in wound healing and anti-aging skin care products [67]. *G. lucidum* polysaccharides attenuated skin flap ischemia-reperfusion injury via thioredoxin-1-dependent antioxidant and anti-apoptotic pathway [74]. The administration of *G. lucidum* polysaccharide into mice skin flap undergoing ischemia-reperfusion injury exhibited increased SOD activity, reduced level of malondialdehyde, and subsequent reduction of the apoptotic keratinocytes and fibroblasts. While the depleted level of thiorexin-1, an endogenous redox signaling regulator, was observed in ischemia-reperfusion flaps, administration of *G. lucidum* polysaccharides restored the level of thiorexin-1 and inhibited subsequent apoptosis signal-regulating kinase 1 (ASK-1)–mitogen-activated protein kinase (MAPK) signaling pathway. *G. lucidum* polysaccharides also protect fibroblasts against UVB-induced photoaging by reducing the ROS levels [75]. UVB-irradiated fibroblasts treated with *G. lucidum* polysaccharides showed increased cell viability, decreased number of β-galactosidase-positive fibroblasts, increased expression of c-telopeptides of type I collagen, decreased matrix metalloproteinase (MMP)-1 expression, and reduced level of intracellular ROS compared with the control.

#### 3.3.2. Epidermal Stem Cell Activation

We investigated the antioxidant activity of *G. lucidum* and its effect on epidermal stem cells [7]. At high concentration (100 μg/mL), *G. lucidum* showed a good antioxidant activity, comparable to ascorbic acid without keratinocyte toxicity. Skin equivalents treated with *G. lucidum* had cuboidal epidermal basal cells with higher proliferative potential compared with control. The expression of integrin α6 and β1 along the basement membrane was upregulated in the *G. lucidum*-treated skin equivalents. (Figure 1) The number of p63- and PCNA-positive cells was increased in skin equivalents treated with *G. lucidum*. Thus, by altering the integrin expression, the antioxidant *G. lucidum* provides favorable stem cell niche in the skin, increasing the stemness and proliferative potential of epidermal basal cells.

### 3.4. Rhodiola

#### 3.4.1. Antioxidant Activity

The genus *Rhodiola* consists of more than 200 species. Of them, approximately 20 species, including *R*. *rosea*, *R*. *sachalinensis*, *R*. *crenulata*, *R*. *sacra*, *R*. *alterna*, *R*. *brevipetiolata*, *R*. *kirilowi*, and *R*. *quadrifida* have been used in traditional medicines for anti-fatigue, anti-depressant, and anti-inflammatory drugs in Asia [76]. *R. rosea*, the most extensively studied species among the *Rhodiola* plants, has pharmacological effects such as elongation of lifespan, stimulation of central nervous system, elevation of work performance, protection on cardiovascular system, nervous system and liver, and increased resistance against bacterial and virus [76]. *Rhodiola* contains a high percentage—up to 41.4%—of polyphenols, such as flavonoids, proanthocyanidines, tyrosol, and cinnamyl alcohol, which might provide its antioxidant property [77,78]. Major components of *Rhodiola* plants are rosavin (rosarian, rhodionin, rhodiosin, and rosin), cinnamyl alcohol, salidroside, and tyrosol [76]. Of them, salidroside and its aglycone, tyrosol, are the most important compounds of *Rhodiola*, and the quality of crude drugs of *Rhodiola* depends on the amount of these two compounds [76]. 

*R. imbricate* extract exhibited antioxidant activity and reduced lipid peroxide levels in the rat excision wound model [79]. *R. imbricate* extract accelerated the process of wound healing as shown by increased rate of wound contraction, decreased time taken for epithelialization, and increased cellular proliferation and collagen synthesis compared with control. *R. rosea* extract was shown to have a high potential for scavenging singlet oxygen and hydrogen peroxide, reducing ferric iron (Fe^3+^), chelating ferrous iron (Fe^2+^) and protecting protein thiol [78]. In cultured human keratinocytes exposed to oxidative stress, *R. rosea* root extract upregulated the cellular antioxidant processes, such as SOD and catalase activities, while it downregulated the glutathione levels, glyceraldehyde-3-phosphate dehydrogenase activity, and thiobarbituric acid reactive substances levels [80]. Oligomeric proanthocyanin from *R. rosea* enhanced the activities of SOD and glutathione peroxidase, and lowered malondialdehyde level in serum, heart, liver and brain tissues in mice [81]. *R. crenulata* root extract protected human skin and human keratinocytes cell line (HaCaT) cells from ionizing radiation by attenuating oxidative stress, cell apoptosis, and MMP levels [82]. 

#### 3.4.2. Epidermal Stem Cell Activation

We investigated the antioxidant activity of *R. sachalinensis* and its effect on epidermal stem cells [7]. At a high concentration (100 μg/mL), *R. sachalinensis* showed an antioxidant activity comparable to ascorbic acid without keratinocyte toxicity. Skin equivalents treated with *R. sachalinensis* showed increased stemness and proliferative potential, and upregulated expression of integrin α6 and β1 along the basement membrane. (Figure 1) The number of p63- and PCNA-positive cells was increased in skin equivalents treated with *R. sachalinensis*. Therefore, *R. sachalinensis* upregulates the expression of integrins and provides the favorable stem cell niche for epidermal basal cells proliferation.

### 3.5. Resveratrol

#### 3.5.1. Antioxidant Activity

Resveratrol (3,5,4′-trihydroxy-*trans*-stilbene) is a natural polyphenol found in grapes, red wine, and berries [8]. It has been extensively explored in the last decades for a potential therapeutic role against aging as well as in various diseases, including cancers and cardiovascular diseases [83]. Resveratrol has various mechanisms of action including (1) reduction of intracellular ROS, (2) activation of sirtuin 1 (SIRT1), a histone deacetylase that increases DNA stability, prolongs survival in mammals, and provides cellular protection against UV radiation through p53 and c-Jun n-terminal kinase (JNK) pathway modulation, (3) significant cancer chemopreventive potential. Resveratrol could also exert its effect via [^3^H]-resveratrol specific binding sites in HaCaT cells [84]. Extensive researches on resveratrol have been carried out in the fields of cosmetology and dermatology to demonstrate its anti-melanogenic and antioxidant properties [8]. 

It has been shown to directly scavenge hydroxyl radical, nitric oxide, superoxide anion radial, and peroxynitrite [85]. Previously, we have also reported ROS scavenging activity of resveratrol by DPPH assay and normal human melanocytes treated with hydrogen peroxide [86]. Resveratrol lowered intracellular level of ROS in human skin fibroblast exposed to hydrogen peroxide in a dose-dependent manner [87]. Human keratinocytes exposed to particulate matter also showed reduced production of ROS in a dose-dependent manner when treated with resveratrol [88]. Beyond its direct antioxidant properties, resveratrol also activates the endogenous antioxidant pathways via the upregulation of the nuclear factor erythroid 2-related factor (Nrf2) [89]. Resveratrol induces the phosphorylation of Nrf2 via the phosphoinositide 3-kinases/Akt (protein kinase B) pathway and releases Nrf2 from the Kelch-like ECH-associated protein (Keap1) [85]. The nuclear translocation of Nrf2 activates the antioxidant response element (ARE), inducing endogenous antioxidant enzymes, such as NADPH quinone dehydrogenase-1 and glutathione peroxidase-2 in normal human keratinocytes [89]. 

Resveratrol decreased apoptosis of HaCaT cells exposed to sodium nitroprusside that served as a donor of nitric oxide free radical [84]. Resveratrol suppresses the overproduction of nitric oxide, which is relevant to premature skin aging occurring upon chronic UV exposure [90]. Resveratrol protects keratinocytes against the UV- or cigarette smoke-induced oxidative damages [91,92]. In cultured keratinocytes, activation of SIRT1 by resveratrol suppresses UVB- and hydrogen peroxide-induced cell death via inhibition of ROS-mediated JNK activation and p53 acetylation [93]. SIRT1 is also involved in the activation of AMP-activated protein kinase (AMPK) [93]. Activation of the AMPK-forkhead box O (FOXO) 3a cascade by resveratrol prevents keratinocytes from oxidative stress-induced senescence and proliferative dysfunction [94]. 

#### 3.5.2. Epidermal Stem Cell Activation

We demonstrated that the antioxidant activity of resveratrol provides favorable stem cell niche in the skin and increases the stemness and the proliferative potential of interfollicular epidermal stem cells [8]. In skin equivalents treated with resveratrol, the expression of integrin α6 was upregulated and the number of p63-positive cells was increased.

### 3.6. Phlorizin (Active Ingredient of Eleutherococcus Senticosus)

#### 3.6.1. Antioxidant Activity

*Eleutherococcus senticosus* (*Acanthopanax senticosus*, also known as Siberian ginseng) is a widely used traditional Chinese herb has been used as an anti-fatigue agent [95]. It grows in Eastern region of Russia and East Asia, including China, Korea, and Japan. Several pharmacological studies on *E. senticosus* has demonstrated its anti-stress, anti-ulcer, anti-irradiation, anti-cancer, anti-inflammatory, and hepatoprotective activities [95]. The leaves of *E. senticosus* inhibit glycosidase to exhibit anti-bacterial properties [96]. Several chemical compounds consisting *E. senticosus* have been reported, including triterpenoid saponins, lignans, coumarins, and flavonoids, among which phenolic compounds are considered to be the most active components [95]. Elutheroside B and E are major active lignands of *E. senticosus*, which have immunomodulatory, antioxidant, and anti-inflammatory activities [95]. Other phenolic compounds such as caffeic acid and chlorogenic acid also showed strong antioxidant activity in vitro and in vivo [97,98]. Water-soluble polysaccharide fractions from *E. senticosus* had a protective effect against oxidative damage exhibiting scavenging activities for hydroxyl radical, superoxide radicals, and DPPH radicals [99]. Extracts of *E. senticosus* showed protective activity against tert-butyl hydroperoxide-induced oxidative stress in rat hepatocytes [100]. *E. senticosus* extracts induced the expression of antioxidant enzymes through the activation of Nrf2, a critical transcription factor that binds to the ARE encoding antioxidant enzymes. 

Phlorizin (phloretin-2′-O-glucoside, various named phloridzin, phlorhizin, phlorrhizin), the main ingredient of *E. senticosus*, belongs to the chemical class of dihydrochalcones [101]. Chalcones (1,3-diaryl-2-propen-1-ones) are flavonoids which display a broad spectrum of bioactivities such as anti-cancer, anti-fungal, anti-bacterial, anti-viral, and anti-inflammatory properties [102]. Dihydrochalcones without α-β double bond comprise phlorectin and its glucoside, phlorizin [103]. Phlorizin is also abundantly present in apples. It has drawn attentions for its pharmacological action to induce renal glycosuria and inhibit intestinal glucose absorption through sodium-linked glucose transporters (SLGTs) [104]. 

Beyond its anti-diabetic activity, the antioxidant activity of phlorizin and its derivatives has been also investigated in vitro [101,105,106]. The potent antioxidant activity of phloretin was shown by peroxynitrite scavenging and inhibition of lipid peroxidation [103]. Phlorizin was shown to prevent the skin from UVB-induced damage in vitro and in vivo by suppressing ROS overproduction, cyclooxygenase-2 (COX-2) expression, and subsequent inflammatory reactions [107]. Inhibition of p38 and JNK-MAPK signaling pathways are involved in the protective effect of phlorizin [107].

#### 3.6.2. Epidermal Stem Cell Activation

**Figure 2:**
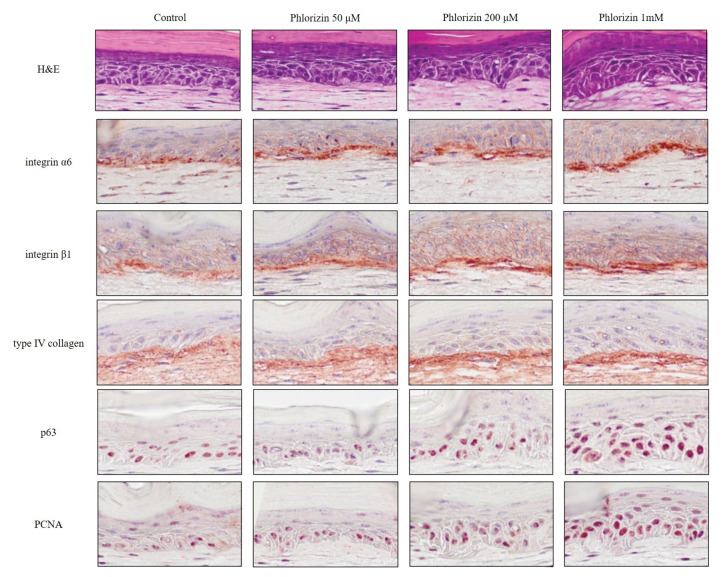
Histological and immunohistochemical findings for phlorizin-treated skin equivalents. Skin equivalents were constructed and incubated in the presence of phlorizin (0, 50, 200 μM or 1 mM). Sections of skin equivalents were stained with hematoxylin and eosin (H&E), and analyzed by immunohistochemical staining for integrin α6, integrin β1, type IV collagen, p63, and PCNA (magnification ×200). (modified from the work by Choi et al. [9]).

Recently, we reported the antioxidant property of *E. senticosus* extracts and phlorizin and its effect on skin equivalents [9]. Skin equivalents treated with 0.002% of *E. senticosus* extracts showed slightly thickener epidermis than control. The expression of p63, PCNA, and integrin α6 was upregulated in skin equivalents treated with *E. senticosus* extracts. Extraction and chromatography indicated that phlorizin is an active ingredient of *E. senticosus* extracts. Compared with ascorbic acid, phlorizin had a relatively weak antioxidant activity at low concentrations (1 mM), but equivalent antioxidant activity at high concentrations (5–10 mM). Skin equivalents treated with phlorizin showed increased thickness of the epidermis, an increased staining intensity for p63, PCNA, integrin α6, and integrin β1. (Figure 2) Linear staining of type IV collagen, a major component of basement membrane, increased in a dose-dependent manner in the phlorizin-treated skin equivalents. We also revealed that phlorizin downregulated the level of miR135b, which is involved in the proliferative potential of epidermal stem cells by regulating the type IV collagen synthesis [27]. In conclusion, *E. senticosus* extracts and their active ingredient, phlorizin, produce a favorable stem cell niche for the epidermal stem cell survival and proliferation via regulating ECM proteins or transmembrane proteins.

### 3.7. Human Tripeptide “Copper-GHK”

#### 3.7.1. Antioxidant Activity

Peptides are amino acids chains that are involved in various cellular processes [108]. The human tripeptide glycyl-L-histidyl-L-lysine (GHK) is an extracellular matrix-derived peptide with a high affinity for copper that forms the copper-GHK complex [109]. In the copper-GHK complex, Cu^2+^ ion binds to the nitrogen from the imidazole side chain of histidine, another nitrogen from the α-amino group of glycine, the deprotonated amide nitrogen of the glycine-histidine peptide bond, and the oxygen from the carboxyl group of the lysine from the neighboring complex [109,110]. The concentration of GHK is associated with age, which is present at 200 mg/L in age of 20–25, and at 80 mg/L by the age of 60–80 [109]. Isolated from human plasma in 1973, it was shown to promote the proliferation of hepatoma cells and the survival of normal hepatocytes by providing copper that is necessary for the cellular functions [111]. Subsequent research has found its beneficial actions in skin, nervous tissue, intestine, bone, and blood vessels [109]. It regulates the wound healing and tissue remodeling process via its antioxidant, anti-inflammatory, and regenerative properties [112]. 

Since the activity of endogenous antioxidants such as SOD1 depends on copper ion, copper deficiency could result in reduced antioxidant enzyme activity and increased oxidative tissue damage [113,114]. Copper-GHK upregulates endogenous antioxidants, supposedly by supplying copper necessary for its function [115]. In rats treated with copper-GHK, the level of SOD and catalase was upregulated during the skin wound healing process [115]. The level of hydroxyl and peroxyl radicals was diminished in the cells treated with copper-GHK [116]. Copper-GHK also reduces oxidative tissue damage by inhibiting iron release and lipid peroxidation in damaged tissues [117]. The presence of iron complexes in damaged tissues increases lipid peroxidation, and thus delays wound healing. Copper-GHK inhibits the release of ferritin iron, probably by binding to the ferritin channels involved in the iron release [117]. 

#### 3.7.2. Regulation of Gene Expressions

In addition to its antioxidant activity, copper-GHK could regulate a large number of human genes. Copper-GHK reduced the transcription of RNA of 54 genes overexpressed in patients with aggressive colorectal cancer. [118]. It also regulates human genes such as *SIGMAR1*, *EPM2A*, *NAIP*, *FGFR2*, and *SLIT2* that are involved in the development and maintenance of nervous system [109].

#### 3.7.3. Regulation of ECM Production

Several studies have suggested copper-GHK as a potential activator of wound repair. Copper-GHK upregulated the synthesis of type I collagen in fibroblast cultures [119]. In an in vivo study using a rat experimental wound, the injection of copper-GHK stimulated collagen synthesis and the expression of type I and III collagen mRNAs [120]. Glycosaminoglycans (dermatan sulfate, chondroitin sulfate) and proteoglycan (decorin), which interact with collagen to organize and strengthen the fibrillary network, were also increased in vitro and in vivo after treated with copper-GHK [120,121,122]. Glycosaminoglycans and proteoglycans are also involved in several wound healing processes, including cell adhesion, migration, and proliferation [123]. Furthermore, copper-GHK alters the expression of MMPs, extracellular proteinases involved in the degradation of ECM required to eliminate damaged and provisional tissue, and to promote new vessel formation and cell migration, during the wound healing process [124]. Several types of MMPs are selectively expressed when wound healing occurs [124]. In dermal fibroblast cultures incubated with copper-GHK, the expression of MMP-2 and tissue inhibitor of metalloproteinase (TIMP)-1 and 2 was increased [125]. Copper-GHK injection induced the prolonged expression of MMP-9 and the higher level of MMP-2 than control, which resulted in the enhanced wound repair in vivo [124]. Taken together, copper-GHK not only stimulates the production of connective tissue but also regulates the remodeling of ECM by modulating MMPs and TIMPs during the wound healing process that requires a rapid ECM turnover. 

#### 3.7.4. Epidermal Stem Cell Activation

We further investigated the effect of copper-GHK on basal keratinocytes [10]. In the monolayer keratinocyte culture, copper-GHK increased the keratinocytes proliferation in a dose-dependent manner in the skin equivalent model. Skin equivalents showed cuboidal-shaped epidermal basal cells with the addition of copper-GHK. Immunohistochemical analysis revealed increased number of PCNA- and p63-positive basal cells. The expression of integrin α6 and β1 was upregulated to a greater degree in the copper-GHK-treated skin equivalents than in the control. Therefore, copper-GHK may increase the survival and proliferation of interfollicular epidermal stem cells by regulating the expression of ECM proteins such as integrin α6 and β1. 

### 3.8. Tripeptide “ACQ: Alanine-Cysteine-Glutamine”

Glutathione is a major endogenous antioxidant within the cells, not only by directly neutralizing free radicals and ROS, but also by inducing the active forms of exogenous antioxidants such as vitamins C and E [126]. Glutathione is a tripeptide with a gamma peptide linkage between the carboxyl group of the glutamate sidechain and the amine group of cysteine, of which cysteine moiety provides the antioxidant property of glutathione [126]. 

**Figure 3:**
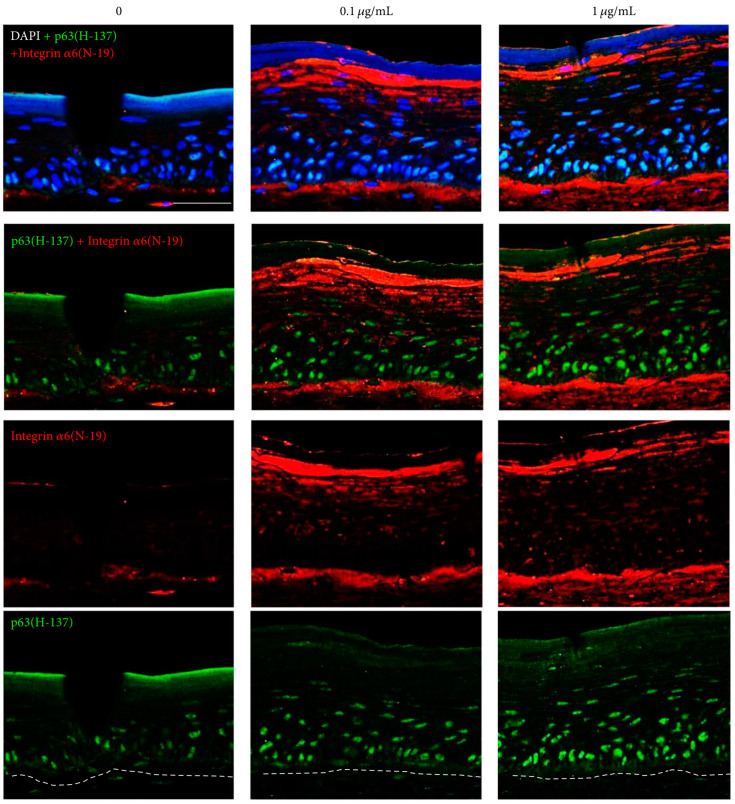
Immunohistochemical staining for integrin α6 and p63 in alanine-cysteine-glutamine (ACQ)-treated skin equivalents. The expression of integrin α6 and the number of p63-positive cells were increased significantly in skin equivalents incubated in the presence of ACQ (magnification ×200). (in the courtesy of Choi et al. [11]).

In an effort to find a novel short bioactive antioxidant peptide that allows transcutaneous delivery, we screened peptide library that contains cysteine. We found a novel tripeptide alanine-cysteine-glutamine (ACQ) that has more significant antioxidant property compared with that of glutathione [11]. ACQ protected fibroblasts and keratinocytes from the hydrogen peroxide-induced oxidative stress. The skin protective effect of ACQ against UV irradiation was demonstrated in BALB/c nude mice fed with 0.1% ACQ solution for 2 or 4 weeks, which had thick epidermis and rare apoptotic cells after UV irradiation. DCF staining revealed lower free radical status and higher number of p63-positive cells in the skin of ACQ fed mice than in the skin of control mice. Skin equivalents treated with ACQ showed increase in the epidermal thickness in dose-dependent manner. Confocal microscopic examination revealed increased number of p63-positive cells and upregulated expression of integrin α6 in the ACQ-treated skin equivalents. (Figure 3) Therefore, ACQ is a good antioxidant tripeptide that increases proliferative potential of the epidermal stem cells via upregulation of integrin α6. 

### 3.9. Hyaluronic Acid

#### 3.9.1. Antioxidant Activity

Hyaluronic acid is a natural polysaccharide that is consisted of glucuronic acid and *n*-acetylglucosamine repeats via β-1, 4 linkage. Repetitive disaccharide units of *n*-acetyl-d-glucosamine and d-glucuronic acid linked by β (1,4) and β (1,3) glycosidic linkages, respectively, constitute the linear structure of hyaluronic acid [127]. Hyaluronic acid is an essential constituent of ECMs in most mature tissues of vertebrates. The largest quantity of hyaluronic acid appears in skin tissue (7–8 g per average adult human) [127]. Due to its biophysical characteristics including biodegradability, biocompatibility, non-toxicity, and non-immunogenicity, hyaluronic acid has been used in biomedical fields such as osteoarthritis surgery, plastic surgery, ophthalmologic surgery, and tissue engineering [127]. 

Several reports have demonstrated the antioxidant properties of hyaluronic acid. In a rat model of carbon tetrachloride-induced liver injury, injection of hyaluronic acid reduced serum alanine aminotransferase (ALT) and aspartate aminotransferase (AST) rise and lipid peroxidation, and increased SOD and glutathione peroxidase activities [128]. High molecular weight hyaluronic acid (HMWHA) with average molecular weight of 5400 kDa and 2000 kDa attenuated DNA damage in leukocytes during oxidative burst via reducing intracellular level of reactive oxidants [129]. The mechanisms for ROS reduction of HMWHA are not completely understood. Hydroxyl functional groups in HMWHA could possibly absorb ROS [130]. HMWHA could interact with the CD44 receptor to activate pathways that regulates cellular redox status and intracellular ROS generation [131]. Endocytosed polyanionic hyaluronic acid molecules through the binding to the CD44 receptor of monocytes and granulocytes chelate Fe^2+^ and Cu^2+^ anions to suppress the generation of hydroxyl radical [129]. 

It was also reported that low molecular weight hyaluronic acid (LMWHA, 200–230 kDa) that is able to penetrate the skin prevents ROS damage in granulation tissue and promotes incisional wound healing in a rat experimental model [132,133]. Irradiation of gamma rays to the native hyaluronic acid increased its antioxidant activity by reducing the molecular weight [134]. Two LMWHAs, LMWHA-1 (145 kDa) and LMWHA-2 (45.2 kDa), inhibit lipid peroxidation and scavenge hydroxyl radical 1,1-diphenyl-2-picryldydrazyl radical superoxide anion in vitro [135]. Its antioxidant and free radical scavenging properties were superior to that of the native hyaluronic acid of 1050 kDA. Administration of LMWHA increased the activity of SOD, catalase, glutathione peroxidase, and total antioxidant capacity in cyclophosphamide-induced immunosuppressed mice [135]. However, the mechanism of antioxidant effect of LMWHA needs further study. 

#### 3.9.2. Epidermal Stem Cell Activation

We investigated the effect of oligosaccharides of hyaluronic acid (400–2000 Da) on epidermal stem cells [12]. Skin equivalents showed increased epidermal thickness in addition of oligosaccharides of hyaluronic acid. Contrary to the control, oligosaccharides of hyaluronic acid-treated skin equivalents showed positive staining for filaggrin in the granular layer. Filaggrin, which is expressed in the granular layer of epidermis during terminal differentiation, is a late marker of keratinocyte differentiation [136]. Thus, the result indicates that oligosaccharides of hyaluronic acid promotes differentiation as well as proliferation of epidermal keratinocytes. The number of p63-positive cells was greater in the basal layer of oligosaccharides of hyaluronic acid-treated skin equivalents than control, indicating the increased stemness of epidermal stem cells. The staining intensity of integrin α6 and β1 along the dermo-epidermal junction was significantly greater than that of control. Our findings indicate that oligosaccharides of hyaluronic acid affect the survival and differentiation of epidermal stem cells probably by altering the expression of ECM proteins such as integrins α6 and β1.

## 4. Application in Skin Rejuvenation 

For the application of the antioxidants in the field of cosmetology and dermatology, further studies may be necessary to determine the proper concentration of the antioxidants in cosmetic products without safety problem. Though it is widely accepted that antioxidants play a role in preventing skin cancer, the risk of carcinogenesis must be strictly evaluated before the application of the antioxidants [137,138]. Searching for optimized formulation or delivery system is also important to enhance the permeation of the ingredients into skin. For example, ascorbic acid has limited application in the cosmetic field due to the unstable structure and low skin penetration [139]. The chemical modification of the hydroxyl group at the C-2 position of ascorbic acid produced stable derivatives such as 2-O-α-D glucopyranosyl-L-ascorbic acid (ascorbic acid 2-glucoside) [140,141]. Conjugated form of palmitoyl-KVK and ascorbic acid (palmitoyl-KVK-L-ascorbic acid) to enhance stability and skin penetration of ascorbic acid not only increased type I collagen synthesis in dermal fibroblasts but also improved facial wrinkles and pigmentation in vivo [142,143]. Ascorbic acid encapsulated into negatively charged liposomes exhibited increased stability, skin permeation [139]. Type I collagen production by fibroblasts and regeneration of UVA-induced keratinocyte damage were demonstrated in vitro.

Similarly, the natural form of resveratrol is chemically unstable to be used in cosmetic formulations [144]. As an approach to enhance stability of resveratrol in cosmetic formulations, resveratrol was acetylated to resveratryl triacetate as a prodrug form [145]. Resveratryl triacetate, which is converted into resveratrol through the action of endogenous esterases, has higher stability in solutions, lower cytotoxicity, and comparable anti-melanogenic effect in cultured melanocytes when compared with resveratrol [145]. The efficacy and safety of 0.4% resveratryl triacetate cream in treating hyperpigmented spots was also demonstrated in a clinical trial [146]. The 0.4% resveratrol triglycolate cream, another derivative of resveratrol esterified with glycolic acid, also showed efficacy in reducing hyperpigmentation in human skin without adverse skin reactions [144].

We previously reported clinical application of the antioxidant solution containing copper-GHK, oligosaccharides of hyaluronic acid, and *Rhodiola* extract (GHR formulation) using hydroporation technique in the treatment of periorbital fine wrinkles and melasma [147,148,149]. Hydroporation technique utilizes subsonic flow of air and the microdroplets of the solution into the skin to exfoliate the epidermis gently and to deliver the cosmetic solution into the skin. Weekly treatments for 8 to 12 weeks improved features of aged skin, such as fine wrinkles, pigmentation, and increased blood vessels. Immunhistochemical study revealed the increased staining intensity for type IV collagen and integrin α6 in the dermo-epidermal junction and p63-positive epidermal stem cells, which was consistent with the result of the in vitro studies using skin equivalents [147]. 

## 5. Conclusions 

Identification of new biological mechanisms associated with skin aging enables the discovery of new active ingredients for cosmetics or cosmeceuticals that may recover biological functions affected by aging [150]. One point of view on skin aging is the dysfunction of epidermal stem cells that regulate self-renewal, proliferation, and differentiation of skin. Stem cell-specific microenvironment, which is regulated by intrinsic and extrinsic signals, could determine the function and survival of the stem cells. Redox status is a critical extrinsic signal that affect the microenvironment surrounding epidermal stem cells. By regulating the expression of ECM, the key component of the stem cell niche, ROS affects the proliferative potential of epidermal basal cells. Radiation of UVB, which is the most important environmental factor causing skin photoaging, also reduces antioxidant enzyme activity, increases intracellular ROS levels in the epidermis, and may contribute to the alteration in the epidermal stem cell function. 

This work addresses the issues of oxidative stress associated with skin aging and provides a list of active ingredients to reverse the dysfunction of epidermal stem cells. We overviewed several antioxidants such as ascorbic acid, plant extracts, peptides, and hyaluronic acid that have ROS scavenging activities and protect cells from oxidative stress-induced damages in vitro or in vivo studies. Using the 3D skin equivalent models, we have demonstrated that antioxidants serve as an activator of epidermal stem cells, which leads to increased survival and proliferation of the stem cells. Antioxidants may modulate the microenvironment of epidermal stem cells by altering the production of ECM. Epigenetic mechanisms such as miR135b are involved in the ECM-mediated regulation of basal keratinocyte proliferation. 

## Abbreviations

ALT	serum alanine aminotransferase
AMPK	AMP-activated protein kinase
ASK-1	apoptosis signal-regulating kinase 1
AST	aspartate aminotransferase
ARE	antioxidant response element
COX-2	cyclooxygenase-2
ECM	extracellular matrix
ERK	extracellular signal-regulated kinases
Fe^2+^/α-KGDDs	Fe^2+^- and α-ketoglutarate-dependent dioxygenases
FOXO	forkhead box O
HaCaT	human skin and human keratinocytes cell line
HDAC	histone deacetylase
HMWHA	high molecular weight hyaluronic acid
iPSC	induced pluripotent stem cell
JHDMs	Jumonji C domain-containing histone demethylases
JNK	c-Jun *n*-terminal kinase
Keap1	Kelch-like ECH-associated protein
LMWHa	low molecular weight hyaluronic acid
MAPK	mitogen-activated protein kinase
MMP	matrix metalloproteinase
NADPH	nicotinamide adenine dinucleotide phosphate
Nrf2	nuclear factor erythroid 2-related factor
PCNA	proliferating cell nuclear antigen
ROS	reactive oxygen species
SIRT1	sirtuin 1
SLGT	sodium-linked glucose transporter
SOD	superoxide dismutase
TET	ten-eleven translocation
TIMP	tissue inhibitor of metalloproteinase
TNF-α	tumor necrosis factor-α
UV	ultraviolet
